# Fully transparent high performance thin film transistors with bilayer ITO/Al-Sn-Zn-O channel structures fabricated on glass substrate

**DOI:** 10.1038/s41598-017-01691-7

**Published:** 2017-05-04

**Authors:** Yingying Cong, Dedong Han, Junchen Dong, Shengdong Zhang, Xing Zhang, Yi Wang

**Affiliations:** 1 Institute of Microelectronics, Peking University, Beijing, 100871 China; 2 Shenzhen Graduate School, Peking University, Shenzhen, 518055 China

## Abstract

In this work, fully transparent high performance double-channel indium-tin-oxide/Al–Sn–Zn–O thin-film transistors (ITO/ATZO TFTs) are successfully fabricated on glass by radio frequency (RF) magnetron sputtering. The ITO layer acts as the bottom channel layer to increase the channel carrier concentration. The top ATZO channel layer, which is deposited via high oxygen partial pressure in the sputtering process, is useful to control the minimum off-state current. After annealing, the ITO/ATZO TFT demonstrates outstanding electrical performances, including a high ON/OFF current ratio (I_on_/I_off_) of 3.5 × 10^8^, a steep threshold swing (SS) of 142.2 mV/decade, a superior saturation mobility (μ_sat_) of 246.0 cm^2^/Vs, and a threshold voltage V_T_ of 0.5 V. The operation mechanisms for double-channel structures are also clarified.

## Introduction

Transparent electronics is becoming a hot research topic, and the metal oxide thin film transistor is one of the key technologies^1, 2^. Owing to the high mobility, low-temperature processing, transparency, and lost cost, metal oxide thin-film transistors (TFTs) have become a promising candidate for driving Active-matrix organic light emitting diode (AMOLED) display panels and other high-performance display applications^3–6^. A series of the novel materials have been recommended as the channel materials to optimize the performances of the TFTs, including In-Ga-Zn-O (IGZO)^7–9^, Al-Zn-O^10^, Hf-In-Zn-O (HIZO)^11^, Al-Sn-Zn-In-O^12^, and so on. We have reported high electrical performances TFTs with Al-Sn-Zn-O (ATZO) channel materials, as the element Sn^4+^ is helpful to increase the material mobility for the special electronic configuration of (n − 1)d^10^ns^0^ (n ≥ 4)^13^ and the element Al enhances the chemical bonds with oxygen^14^ and improves the electrical characteristics at low temperature^15^.

Based on the novel ATZO TFT, modifying the device structures is an effective and convenient method to further improving the electrical performances. For example, we have improved the electrical performances of the ATZO TFTs with the double-channel structures in a previous work^16^. No complicated process is added to the fabrication flow. The double-channel structures are grown by successively depositing bilayer ATZO films with different oxygen partial pressures in the sputtering process. However, with the same kind of the channel materials, the double channel ATZO TFTs just keep the better performances of the single-channel TFTs without further improvements. Therefore in this letter, we adopt the indium-tin-oxide (ITO) material as the bottom channel layer to increase the channel carrier concentration and maximum channel current. The ATZO layer with higher channel resistivity is used as the top channel layer to control the minimum off-state current. Double-channel ITO/ATZO TFTs with excellent performances are achieved, and the operation mechanisms for double-channel structures are clarified.

## Results and Discussion

**Figure 1:**
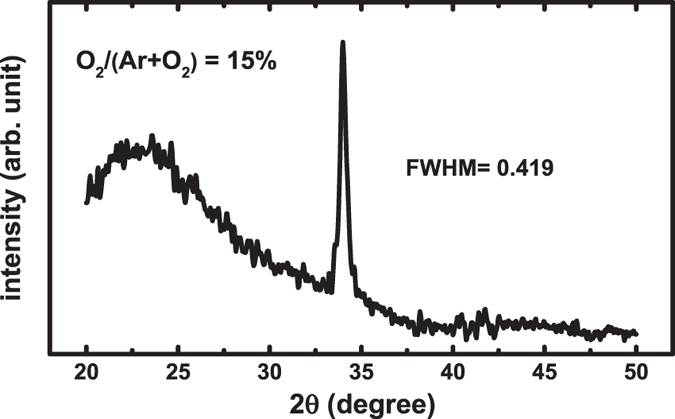
XRD scan spectra of the ATZO films with 15% oxygen partial pressure. The thickness of the films is 40 nm.

The XRD spectra of the ATZO film with 15% oxygen partial pressures are shown in Fig. 1. The test sample was fabricated via the simultaneous deposition of channel layers in the sputtering chamber on glass. The spectra show that the ATZO film is polycrystalline in the hexagonal wurtzite structure with the preferred c-axis orientation. The extracted full width half maximum (FWHM) of the ATZO sample is 0.419. The grain size of the film is calculated by the Scherrer formula:

1 $${\rm{D}}={\rm{\kappa }}{\rm{\lambda }}/{\rm{\beta }}\,\cos \,{\rm{\theta }}$$

where, D is the mean grain size, κ = 0.9 is the dimensionless shape factor, λ is the X-ray wavelength, β is equal to the FWHM, and θ is the diffraction angle. The estimated grain size of the ATZO film is 19.5 nm.

**Figure 2:**
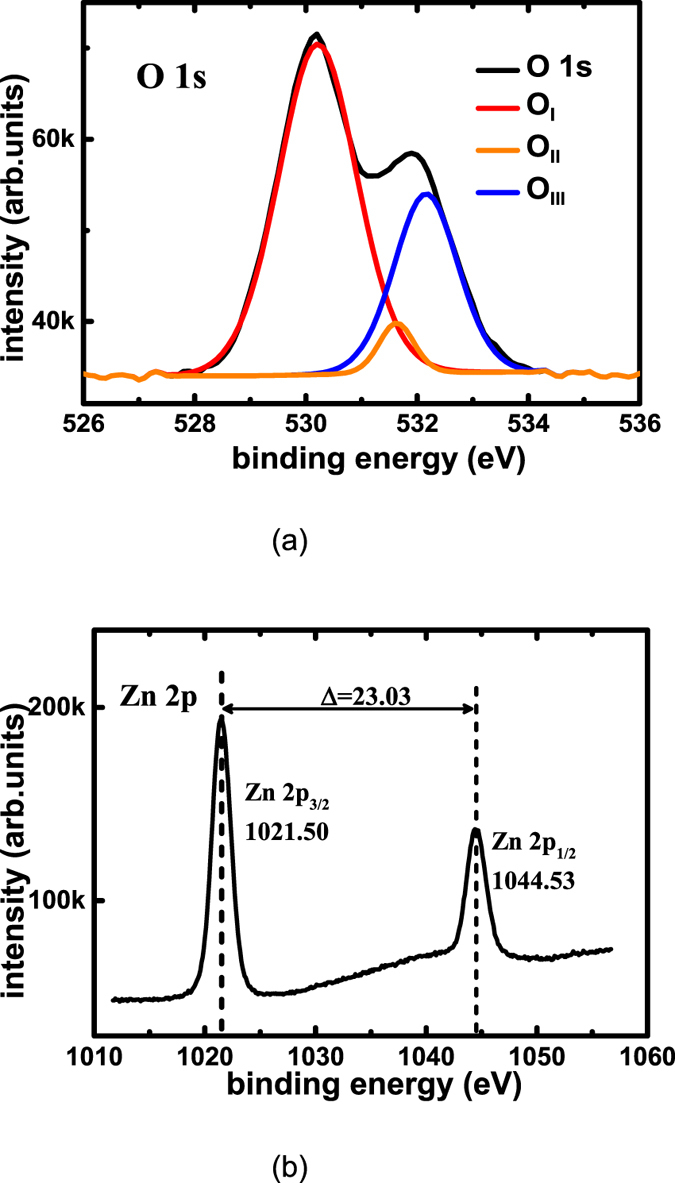
XPS scan spectra of ATZO films. (**a**) O 1 s and (**b**) Zn 2p spectra of the ATZO films with oxygen partial pressure of 15%.

The O 1s XPS scan spectra of the ATZO film are shown in Fig. 2(a). To clarify the oxygen chemical bonds in the ATZO films, the asymmetric O 1 s peaks are divided into three peaks, which are centered at 530.2 eV (O_I_), 531.6 eV (O_II_), and 532.2 eV (O_III_). The O_I_, O_II_, and O_III_ peaks are generally attributed to O^2−^ bonded by metal ions (Zn–O, Al–O, and Sn–O), O^2−^ in the oxygen-deficient region (such as V_O_), and chemisorbed oxygen at grain boundaries or the surface of the film (such as metal hydroxide and oxy-hydroxide oxygen), respectively^17, 18^. The relative quantities of the lattice oxygen, the V_O_, and surface hydroxyl group can be achieved from the peak area ratio of the three peaks (O_I_, O_II_, and O_III_). The relative O_II_ area ratio of the ATZO film is 4.47%. Figure 2(b) shows the Zn 2p peaks of the ATZO film. Due to the spin orbit split, the Zn 2p spectra have Zn 2p_1/2_ and 2p_3/2_ peak centered at 1021.50 eV and 1044.53 eV, respectively. It is generally accepted that the Zn 2p_3/2_ peak centered at 1021.4 eV represents Zn interstitial in the oxygen-deficient regions^19^. The Zn interstitial and the V_O_ generate free carriers in n–type metal–oxide semiconductors.

**Figure 3:**
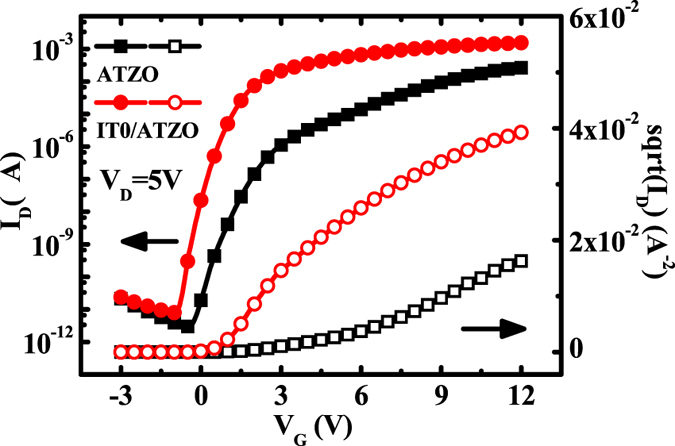
Transfer characteristics of the single-channel ATZO and double-channel ITO/ATZO TFTs. V_D_ = 5 V.

**Figure 4:**
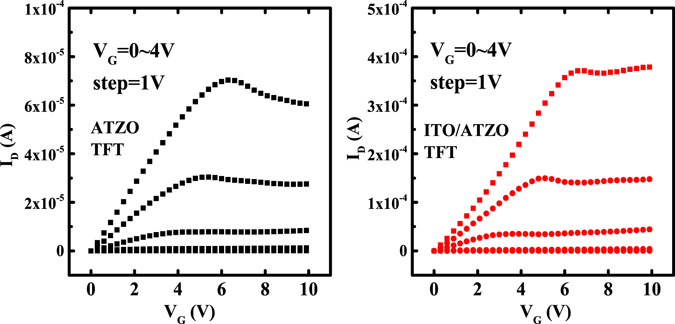
Output characteristics of (Left) single-channel ATZO and (Right) double-channel ITO/ATZO TFTs.

Figure 3 shows the transfer curves of the single-channel ATZO and double-channel ITO/ATZO TFTs. Because the high oxygen partial pressure suppresses the oxygen vacancies in the channel film, the ATZO channel has low carrier concentrations. The single-channel ATZO TFT demonstrates a low off-state current (I_off_), a low on-state current (I_off_), an on-to-off current ratio (I_on_/I_off_) of 8.9 × 10^7^, a V_T_ of 2.9 V, and a large subthreshold swing (*SS*) of 356.6 mV/dec. The extracted μ_sat_ is 26.6 cm^2^/Vs. To enhance the electrical characteristics of single-channel ATZO TFT, an ITO layer is inserted between the gate insulator and the ATZO channel and acts as the bottom channel. Due to the high-conductivity ITO film, the device performances is improved significantly compared with the single-channel ATZO device. The I_on_ obviously increases and the maximum value is an order of magnitude higher than that of the single-channel ATZO TFT. The V_T_ shifts to the left axis due to the increased carrier concentration in the total channels. The I_off_ increase slightly and the SS becomes steeper. The output curves of the single-channel ATZO and double-channel ITO/ATZO TFTs are shown in Fig. 4, respectively. Both the devices demonstrate n-type transistor characteristics.

**Figure 5:**
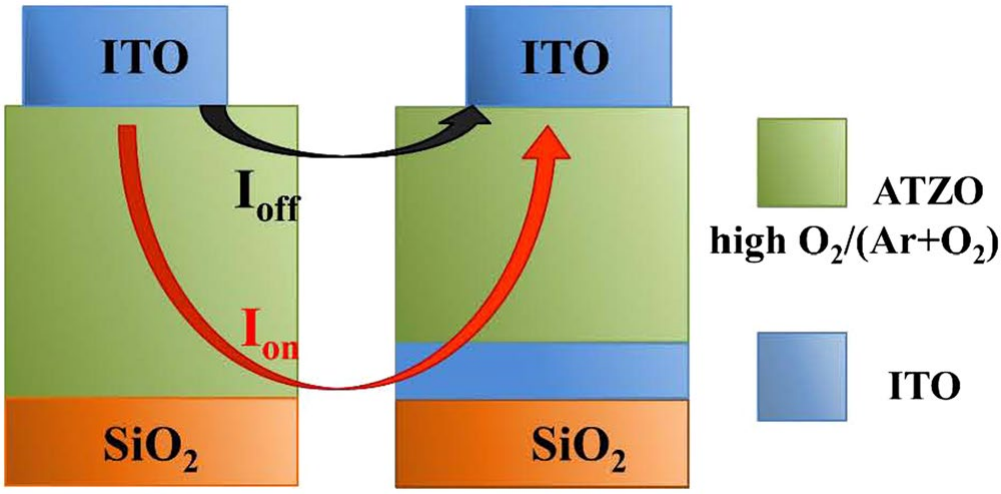
Schematic operation mechanism for (Left) single-channel ATZO and (Right) double-channel ITO/ATZO TFTs.

The operation mechanism for double-channel TFTs can be explained by the schematic current paths shown in Fig. 5. In the on-state, the bottom ITO channel offers more free electrons and decreases the total channel resistance, which increases I_on_ and μ_sat_ and decreases V_T_, respectively. In the off-state, because the thick top ATZO channel layer with higher resistance controls the off-state performances, the double-channel ITO/ATZO TFT demonstrates a low I_off_. Therefore, the I_off_ does not increase as much as the I_on_, which is not influenced by the addition of high-conductivity ITO film. Thus, the ITO/ATZO TFT demonstrate a superior I_on_/I_off_ of 1.9 × 10^8^ and the electrical performances of the ITO/ATZO TFT provides a clear evidence for the operation mechanism of the double-channel TFTs.

**Figure 6:**
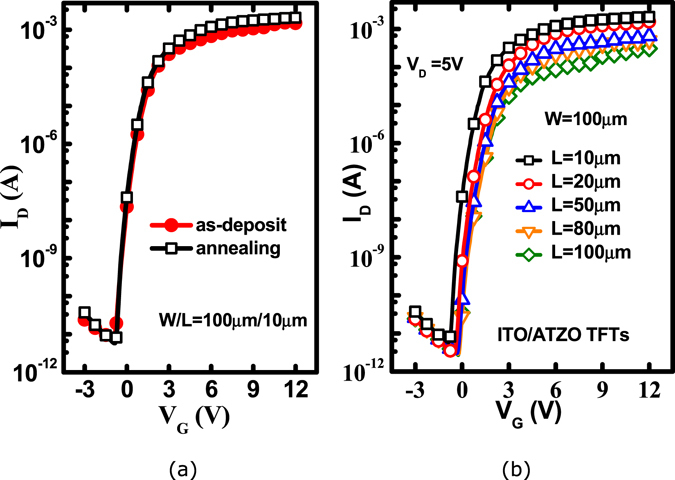
(**a**) Comparison of the transfer curves of the device after annealing. (**b**) Transfer characteristics of the double-channel ITO/ATZO TFTs with various width-to-length ratios.

Figure 6(a) shows the changes in the electrical performances of the double-channel ITO/ATZO TFT after thermal annealing in a vacuum at 150 °C for 30 min, V_D_ = 5 V. The device demonstrates superior performances after annealing. The I_on_ increases and I_off_ decreases slightly. The I_on_/I_off_ increases to 3.5 × 10^8^. The μ_sat_ is 246.0 cm^2^/Vs and the V_T_ is 0.5 V. The subthreshold region becomes much steeper and the SS is 142.2 mV/dec. The transfer characteristics of the ITO/ATZO TFTs with various width-to-length ratios after thermal annealing are shown in Fig. 6(b). All the devices demonstrate good electrical performance. As the channel length decreases, the I_on_ gradually increases and V_T_ shifts to the negative axis.

In conclusion, we successfully fabricate double-channel ITO/ATZO TFTs. The high-conductivity ITO film is adopted as the bottom channel layer to improve the I_on_ and μ_sat_ of ATZO TFTs and the top ATZO channel layer with higher resistance controls the off-state characteristics. The double-channel ITO/ATZO TFTs shows excellent electrical characteristics, including a high ON/OFF current ratio of 3.5 × 10^8^, a high saturation mobility μ_sat_ of 246.0 cm^2^/Vs, a low threshold voltage (V_T_) of 0.5 V, and a steep threshold swing SS of 142.2 mV/decade. Thus, with a simple fabrication process and superior electrical characteristics, the ITO/ATZO TFTs show promising applications in the high-performance display fields.

## Methods Section

**Figure 7:**
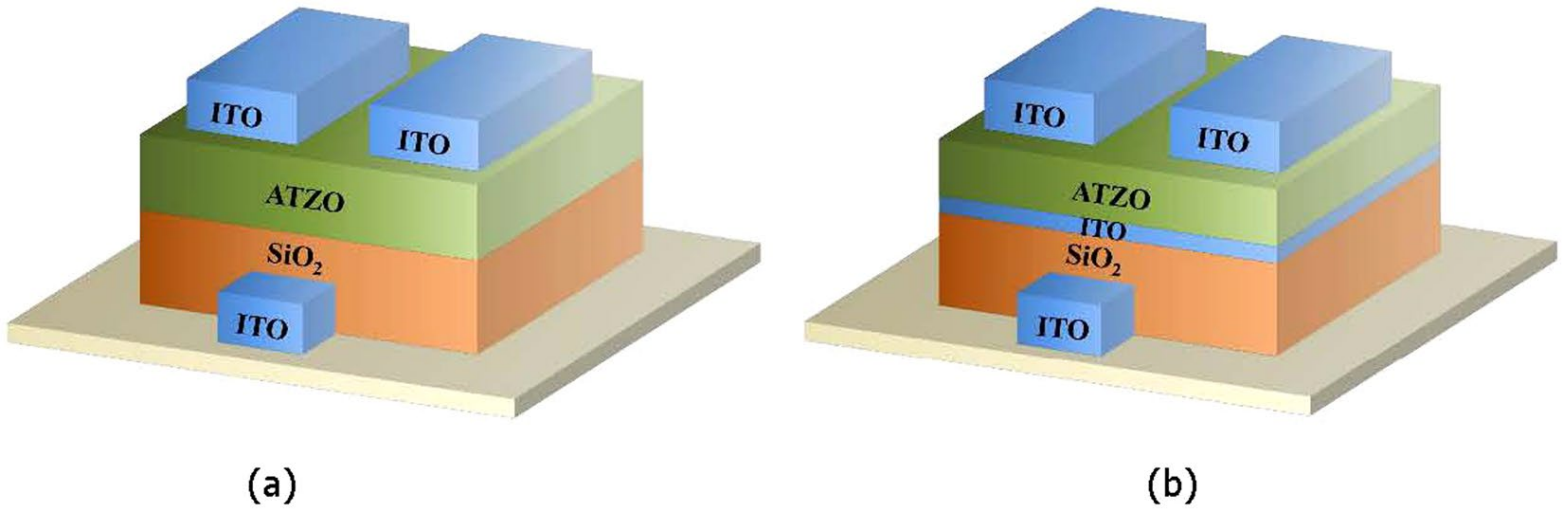
The schematic structures of (**a**) the single-channel ATZO and (**b**) the double-channel ITO/ATZO TFTs.

In this work, the ATZO TFTs were fabricated on glass substrates with a conventional inverted-staggered structure. Briefly the fabrication process isas follows. First, an ITO gate electrode was deposited on glass by RF magnetron sputtering at room temperature with 100 nm film thickness. Second, a 150-nm-thick SiO_2_ film, which was served as the gate insulator layer, was formed by plasma enhanced chemical vapor deposition (PECVD). The deposition temperature was 80 °C. To create better interface properties between the insulator and channel layer, the ATZO channel layer was deposited by RF magnetron sputtering successively after formation of the SiO_2_ film at room temperature. The wt% of SnO_2_ and Al_2_O_3_ in the ATZO sputtering target are both 2%. The oxygen partial pressure [O_2_/(O_2_ + Ar)] in the sputtering chamber was 15% and the channel layer was 40-nm-thick. Finally, an ITO source/drain electrode was deposited using the same process conditions with the gate electrode after formation of the channel layer. For the double-channel structure, we introduced the ITO film as the bottom channel layer between the insulator and ATZO channel layer to optimize the electrical performances. The oxygen partial pressure of the ITO film was 5%. The total channel thickness was 40 nm, including a 5-nm-thick ITO and 35-nm-thick top ATZO channel layer. Figure 7 shows the schematic structures of the single-channel ATZO and the double-channel ITO/ATZO TFTs.

During the fabrication process, we adopted standard photolithography and lift-off technique. The electrical performances of the ATZO TFTs was measured using a semiconductor parameter analyzer (Agilent 4156 C) at room temperature. The chemical states of atoms in the ATZO films were investigated using X-ray photoelectron spectroscopy (XPS, Axis Ultra). The crystal quality conditions of ATZO films were tested using X-ray diffraction (XRD, Rigako).
